# Organ System Network Disruption Is Associated With Poor Prognosis in Patients With Chronic Liver Failure

**DOI:** 10.3389/fphys.2020.00983

**Published:** 2020-08-05

**Authors:** Yen Yi Tan, Sara Montagnese, Ali R. Mani

**Affiliations:** ^1^Network Physiology Laboratory, UCL Division of Medicine, University College London, London, United Kingdom; ^2^Department of Medicine, University of Padova, Padua, Italy

**Keywords:** network physiology, network medicine, cirrhosis, survival, mutual information

## Abstract

**Background:**

A healthy individual has a high degree of functional connectivity between organ systems, which can be represented graphically in a network map. Disruption of this system connectivity is associated with mortality in life-threatening acute illnesses, demonstrated by a network approach. However, this approach has not been applied to chronic multisystem diseases and may be more reliable than conventional individual organ prognostic scoring methods. Cirrhosis is a chronic disease of the liver with multisystem involvement. Development of an efficient model for prediction of mortality in cirrhosis requires a profound understanding of the pathophysiologic processes that lead to poor prognosis. In the present study, we use a network approach to evaluate the differences in organ system connectivity between survivors and non-survivors in a group of well-characterized patients with cirrhosis.

**Methods:**

201 patients with cirrhosis originally referred to the Clinic five at the University Hospital of Padova, with 13 clinical variables available representing hepatic, metabolic, haematopoietic, immune, neural and renal organ systems were retrospectively enrolled and followed up for 3, 6, and 12 months. Software was designed to compute the correlation network maps of organ system interaction in survivors and non-survivors using analysis indices: A. Bonferroni corrected Pearson’s correlation coefficient and B. Mutual Information. Network structure was quantitatively evaluated using the measures of edges, average degree of connectivity and closeness, and qualitatively using clinical significance. Pair-matching was also implemented as a further step after initial general analysis to control for sample size and Model for End-Stage Liver Disease (MELD-Na) score between the groups.

**Results:**

There was a higher number of significant correlations in survivors, as indicated by both the analysis indices of Bonferroni corrected Pearson’s correlation coefficient and the Mutual Information analysis. The number of edges, average degree of connectivity and closeness were significantly higher in survivors compared to non-survivors group. Pair-matching for MELD-Na was also associated with increased connectivity in survivors compared to non-survivors over 3 and 6 months follow up.

**Conclusion:**

This study demonstrates the application of a network approach in evaluating functional connectivity of organ systems in liver cirrhosis, demonstrating a significant degree of network disruption in organ systems in non-survivors. Network analysis of organ systems may provide insight in developing novel prognostic models for organ allocation in patients with cirrhosis.

## Introduction

A network approach in medicine represents a shift from a reductionist approach, which considers involvement of distinct physiological components in the disease process. Although such a reductionistic approach has been fruitful in the development of therapy for Mendelian disorders (e.g., haemophilia), it has failed to uncover the true mechanism of complex disorders such as sepsis and multiple organ failure. By contrast, a network approach works on the basis that these components interact non-linearly to coordinate robust integrated functions (Higgins, 2002).

Network analysis has now been biologically applied on multiple levels, including sub-cellular (e.g., gene expression and protein dynamics) (Arodz and Bonchev, 2015; Kontou et al., 2016; Joehanes, 2018), cellular (e.g., neural networks) (Rothkegel and Lehnertz, 2014; Fernandes et al., 2015; Edwards et al., 2018) and tissue level signaling (Oliveira et al., 2014; Taroni et al., 2017). It is now also being applied to organ system analysis at a functional level. A network approach also has non-biological medical applications, including use in the prediction of evolution of research communities (Yu et al., 2012; Shirazi et al., 2016) and health informatics.

Fundamental research in the emergent modern field of network physiology and network medicine has laid the foundation for understanding and quantifying global physiological behavior that results from networked interactions across systems, coordinated over a range of space and time scales. While this review by Ivanov et al. (2016) presents an overview of the current focus and progress in the field, there is no shortage of work that continues to reinforce the link between physiological coupling, phase transitions and phenotypic network states across our complex physiological systems (Bartsch et al., 2012; Bartsch and Ivanov, 2014; Liu et al., 2015; Lin et al., 2016). Such research has yielded important findings, such as showing that the network in specific physiological states are characterized by specific topology and coupling strength between systems (Bashan et al., 2012).

One of the first applications of a network approach to organ system analysis in complex disorders was proposed by Godin and Buchman (1996), who defined the role of organs as biological oscillators that maintain orderly coupling through system-wide communication networks such as cytokines. They demonstrated the progression of the systemic inflammatory response syndrome to multiple organ dysfunction syndrome, with the impairment of interorgan connectivity being modulated by excessive inflammatory stimuli. While individual organ system dysfunction is well categorized (Marshall et al., 1995), disruption of organ systems has been found to bemortality-associated.

Recently, Asada et al. (2016) reported the first clinical application of network analysis to evaluate interorgan relationships between critically ill surviving and non-surviving patients with multiple organ failure. They challenged the reliability of conventional scoring methods, which sum up degrees of individual organ dysfunction to represent systemic illness pathophysiology and disease severity. Based on their network analysis, the degree of organ system disruption was associated with poor prognosis independently of conventional scoring methods. Survivors consistently yielded a higher number of edges and clusters compared to non-survivors in their organ connectivity network structures. Such a network physiology approach for the early detection of critical illnesses facilitated by big data and novel analytic and computational approaches has merit and is encouraging, even in light of limitations (Moorman et al., 2016). More recently, Asada et al. (2019) followed up exploring the differences in connectivity between specific organ system clusters in critically ill patients. These include the respiratory-renal-inflammatory system cluster and the cardiovascular-hepatic-coagulation system cluster. The study revealed that stability of organ clusters was preserved in survivors as long as organ systems formed an interactive network, regardless of severe dysfunction. In contrast, organ cluster instability and organ system isolation was associated with mortality.

While organ system network analysis has been applied to critical illness of an acute nature, its application to critical illness of a chronic nature has not been investigated. Liver cirrhosis represents an interesting a candidate due to its multiorgan involvement and prevalence of approximately 0.15% (Schuppan and Afdhal, 2008). This is characterized by portal hypertension, ascites formation, hepatorenal syndrome, hepatic encephalopathy, hyperdynamic circulation, cardiomyopathy, autonomic dysfunction and an impaired immune response (European Association for the Study of the Liver, 2010; Licata et al., 2014; Vilstrup et al., 2014). Cirrhosis is hence a multisystemic disease that affects the hepatic, cardiovascular, immune, renal and neurological systems (Schuppan and Afdhal, 2008).

Survival prediction is important in cirrhosis patients, particularly for organ transplant allocation. The Model for End-Stage Liver Disease (MELD) score has been used in recent years to this aim, and has subsequently been refined with the inclusion of serum sodium levels (MELD-Na) (Kamath et al., 2007; Martin and O’Brien, 2015). This score comprizes of indices including serum bilirubin, serum creatinine, serum sodium, and coagulation factors. Recently, physiological markers (e.g., EEG and heart rate variability indices) have been shown to be independent of MELD in predicting survival (Montagnese et al., 2015; Bhogal et al., 2019). Therefore, conventional clinical scores still have room to improve in reflecting the multisystem nature of liver cirrhosis. In the present study, we aim to use a network approach to evaluate the differences in organ system connectivity between liver cirrhosis survivors and non-survivors.

## Materials and Methods

### Participants and Ethics

The study protocol was approved by the Hospital of Padova Ethics Committee. All participants provided written, informed consent. This study was conducted according to the Declaration of Helsinki (Hong Kong Amendment) and Good Clinical Practice (European) guidelines. The study population consists of patients with liver cirrhosis referred to the liver unit, Clinic five of the University Hospital of Padova for assessment of hepatic encephalopathy between 2009 and 2018. The exclusion criteria include patients with hepatocellular carcinoma and patients unconfirmed for hepatocellular carcinoma. Two hundred and one patients (156 males, age ± SD: 57 ± 11 years) met the inclusion/exclusion criteria. On the day of study, 26% patients were classed as Child A, 52% as B, and 22% as C. The average (±SD) MELD score was 14 ± 5 and the average MELD-Na score 15 ± 6.

### Follow-Up Time and Survivors vs Non-survivors

Patients were classified into 3-, 6-, and 12-month follow-up groups, corresponding to a follow-up time threshold of 90, 180, and 360 days. These follow-up groups were then further classified into a survivor group and a non-survivor group.

During each follow-up, the clinical variable measurements and survivor status of patients were updated. Patients who died during the follow-up periods formed the non-survivor group. Patients who survived formed the survivor group.

Patients who had follow-up information less than a follow-up threshold were censored from analysis as their survival status is unconfirmed. For example, if a patient had a follow-up at 150 days which confirmed they were alive, but had no further update beyond that, they would be included in the 3 month follow-up group but censored from the 6 month follow-up group.

### Transplantation

During data collection of the study population, a number of patients underwent successful liver transplantation for liver failure. These patients are considered dead at the time of transplantation.

### Clinical Variables

Clinical and laboratory variables were chosen to represent individual organ or system function. These clinical and laboratory variables were derived retrospectively from a preliminary study using a Random Forest machine learning algorithm (Breiman, 2001). Variables which did not hold any weight in contributing to the outcome of mortality in patients were excluded to ensure that our study focused on the variables that have weight in mortality prediction and are hence included in the analysis in our study. Using this approach redundant clinical variables were eliminated, narrowing down the chosen clinical variables to 13. These include C-reactive protein (CRP), serum albumin, total bilirubin, prothrombin time, international normalised ratio (INR), ammonia, hemoglobin, serum creatinine, serum sodium, ascites and hepatic encephalopathy. Hepatic encephalopathy was classified as (0) unimpaired: no clinical evidence of hepatic encephalopathy and no defining EEG or psychometric abnormalities; (1) minimal hepatic encephalopathy: no clinical evidence of hepatic encephalopathy but abnormal EEG and/or impaired psychometric performance; and (2) overt hepatic encephalopathy: clinically evident neuropsychiatric disturbances (Asada et al., 2019). An addition of the MELD-Na (Elwir and Lake, 2016) and Child-Pugh (Child and Turcotte, 1964; Pugh et al., 1973) scores were calculated. Table 1 indicates the list of clinical variables with their physiological interpretation.

**TABLE 1:** The list of nodes (clinical variables) along with their physiological interpretation and associated major organ system(s) used in this study.

Clinical variable	Physiological interpretation	Major organ system(s)
CRP (C-reactive protein)	An acute phase protein made by the liver that is released into the blood during infection or systemic inflammation	Immune system
Alb (serum albumin)	The most abundant protein in blood which is synthetized by the liver. It has important function in microcirculation. It is a maker of synthetic liver function	Hepatobiliary system and microcirculatory system
Tot Bili (total bilirubin)	Bilirubin is a yellow pigment that occurs in the normal catabolic pathway that breaks down haem in the body. It is excreted by the liver and is marker for excretory liver function	Hepatobiliary system and Hematologic
PTcP (prothrombin time)	A blood test that measures the time it takes for the blood to clot	Coagulation system
INR (International Normalized Ratio)	A blood test that measures the time it takes for the blood to clot	Coagulation system
Ammonia	A metabolic by-product which is eliminated after detoxification in the liver	Hepatobiliary system and Metabolic system
Hb (hemoglobin)	A protein in the red blood cells that carries oxygen	Hematologic system
Creatinine (serum creatinine)	An endogenous compound that is excreted by the kidneys	Renal system
Na (serum sodium)	The major cation in plasma. Its concentration is tightly regulated by renal and endocrine systems	Renal system and Endocrine system
Ascites	Abnormal accumulation of fluid in the abdomen (peritoneal cavity)	Microcirculatory system
HE (hepatic encephalopathy)	Decline in brain function that occurs as a result of severe liver disease	Central nervous system
Pugh (Child-Pugh score)	A scoring system for assessing the severity of chronic liver disease	Hepatobiliary system, Coagulation system and Central nervous system
MELD (model for end-stage liver disease-sodium)	A scoring system for assessing the severity of chronic liver disease	Hepatobiliary system, Coagulation system and Renal system

### Generation of Network Maps

In the network maps, individual clinical variables are represented as nodes. A significant correlation between two nodes is represented by the formation of an edge.

There are many approaches using different indices to evaluate the strength of edges between clinical variables in a network map. This ranges from simple linear approaches such as the Pearson’s correlation coefficient analysis to more complex non-linear approaches such as the information theory Mutual information analysis. In the present study, the approach of using the Pearson’s correlation coefficient with Bonferroni’s correction analysis (Armstrong, 2014) is compared against the approach of using the Mutual information analysis (Steuer et al., 2002; MacKay, 2005). An edge was formed between two clinical variable nodes if the association between two clinical variables was equal to, or greater than the determined cut-off value for the analysis index. All values that did not reach the cut-off threshold were considered zero in the adjacency matrix. For the Pearson’s correlation analysis, the cut-off refers to the correlation values that have passed the Bonferroni significance threshold of 0.0038462, which is the result of an adjusted *p*-value of 0.05 taking into account the number of pairwise comparisons made. For the mutual information analysis, the optimal cut-off value to elucidate informative network connections was determined to be 0.75 after testing a range of values. The use of a lower threshold resulted in an over-saturation of network connections, yielding no interpretable information, while the use of a higher threshold resulted in a lack of network connections.

**FIGURE 1:**
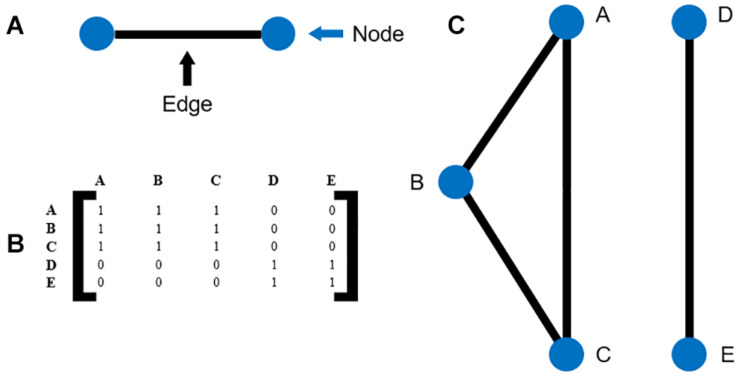
**(A)** Illustration of nodes and edges **(B)** example of an adjacency matrix **(C)** the network map corresponding to the adjacency matrix in **(B)**.

The adjacency matrices for the survivor and non-survivor groups were plotted for the follow-up times of 3, 6, and 12 months as network maps. An example is illustrated in Figure 1. A force-directed graph drawing algorithm was used for network visualization. This algorithm minimizes edge crossings and facilitate clear identification of clusters in the network graph.

### Software Development

All computation and analyses were carried out using MATLAB build R2018b (The MathWorks Inc, 2018). The software developed to compute organ system network analysis requires patient data input in CSV file format. Omitting headings, clinical variables occupy rows and patient data occupies columns. Where pair-matching is utilized, the software generates two equally sized datasets with samples drawn from the original dataset and pair-matched based on the chosen matching criteria. There is an element of randomization where there is more than one possible match to a given sample.

The chosen analysis index is applied and an [*n* × *n*] adjacency matrix is generated, where *n* represents the number of clinical variables. Fully labeled and color distinguished weighted correlation networks of survivors and non-survivors are generated as graph subplots for each follow-up timeframe. The thickness of edges reflects the weight or strength of correlation. The software has been designed to include flexibility of customization of parameters such as node labels, edge widths, node colors, node sizes, edge colors, node layout types and titles.

Furthermore, a quantitative summary table of network parameters including number of edges, degree of connectivity, betweenness and closeness is generated (Freeman, 1978; Valente et al., 2008). An adjacency matrix of shortest paths between nodes is also included.

The written software, which code can be found in this GitHub repository (Tan, 2019), executes the following algorithm:

1. Retrieve CSV format datasets for survivor and non-survivor groups. The option to pair-match datasets with randomization based on an existing column criterion can be selected at this stage (see section: Pair-matching).
2. Execute a correlation analysis selected by user input on the datasets.
3. Output a weighted correlation adjacency matrix.
4. Generate a network map based on this adjacency matrix.
5. Calculate network quantification parameters.

### Pair-Matching

During follow-up timeframes, the number of patients in the survivor and non-survivor groups continually change. As a result, the comparisons made between survivor and non-survivor group datasets drew unequal sample sizes, which could lead to a clearer observed effect in the larger sample size group. Furthermore, there may have been a difference in the severity levels of liver disease between survivor and non-survivor groups resulting in the observed effects. Pair-matching was hence implemented as a further step after initial general analysis to control for sample size and MELD-Na score (McClatchey et al., 1992). The option to pair-match can be included in step 1 of the software algorithm, using the additional pair-matching algorithm outlined in Supplementary Appendix A.

After pair-matching, the original algorithm detailed in the previous software development section will continue executing step 2 to 5. With the removal of the pair-matched criteria column, the number of clinical variables becomes 12. To prevent confusion, non-pair-matched analyses will be termed general analysis.

### Correlation Analysis

Pearson’s correlation is a good measure to evaluate linear correlation between two variables, given the normal distribution of underlying data (Mukaka, 2012). This represents a simplified approach in getting a preliminary, general overview of interorgan relationship in patients. However, it is widely known that biological signaling and regulatory networks are dynamic and complex (Higgins, 2002; Janson, 2012; Blokh and Stambler, 2017). Information theory measures account for the quantity of biological information transmitted independently of network complexity, and are hence robust and sensitive to non-linear relationships and better suited for organ system network analysis (Steuer et al., 2002; Rhee et al., 2012).

Mutual information is one measure that calculates the reduction in the uncertainty of information transmitted between two variables, widely used in network reconstruction and reverse engineering (Basso et al., 2005; Ziv et al., 2007; Iglesias, 2013; Mousavian et al., 2016). It is defined as the difference between the joint entropy of X and Y and the joint entropy under the assumption of independence of X and Y, with the formula (MacKay, 2005):

I(X;Y)=H(X)+H(Y)-H(X,Y)

The higher the mutual information between two variables, the more dependent the two variables are to each other. The mutual information analysis was computed using an external script (Mikhail, 2020). The cut-off for this analysis index was *I*(*X*;*Y*) ≤ 0.75.

### Network Analysis

The differences in organ system interaction between survivor and non-survivor groups was evaluated quantitatively and qualitatively for the follow-up timeframes of 3, 6, and 12 months using the Bonferroni corrected Pearson’s correlation and mutual information analysis indices.

Quantitatively, organ system interaction was evaluated using:

1. Total number of edges. This is the conventional method of comparison for differences in network structure (Tichy et al., 1979; Rowley, 1997).
2. Average degree of connectivity. A node’s degree of connectivity is the total number of edges connected to the node (Freeman, 1978; Valente et al., 2008).
3. Average closeness. A node’s closeness measure is the inverse sum of distances from the node to all other nodes in the network graph and is otherwise a measure of centrality (Freeman, 1978; Valente et al., 2008).

Qualitatively, we attempt to explain the clinical significance of notable organ system interactions and changes observed in the network maps of patients.

## Results

### Patient Groups

The number of patients in the survivor and non-survivor groups over a follow-up timeframe of 3, 6, and 12 months is outlined in Table 2 for general, MELD-Na pair-matched Bonferroni corrected Pearson’s correlation analysis and for MELD-Na pair-matched mutual information analysis.

**TABLE 2:** Number of patients for (A) general analysis, (B) pair-matched correlation analysis, and (C) pair-matched mutual information analysis.

Follow-up time	Survivors	Non-survivors	Total
**(A) General analysis**			
3 months	187	14	201
6 months	141	21	162
12 months	75	31	106
**(B) Pair-matched Bonferroni corrected Pearson’s correlation analysis**			
3 months	11	11	22
6 months	13	13	26
12 months	17	17	34
**(C) Pair-matched mutual information analysis**			
3 months	14	14	28
6 months	13	13	26
12 months	17	17	34

### Network Structure

**FIGURE 2:**
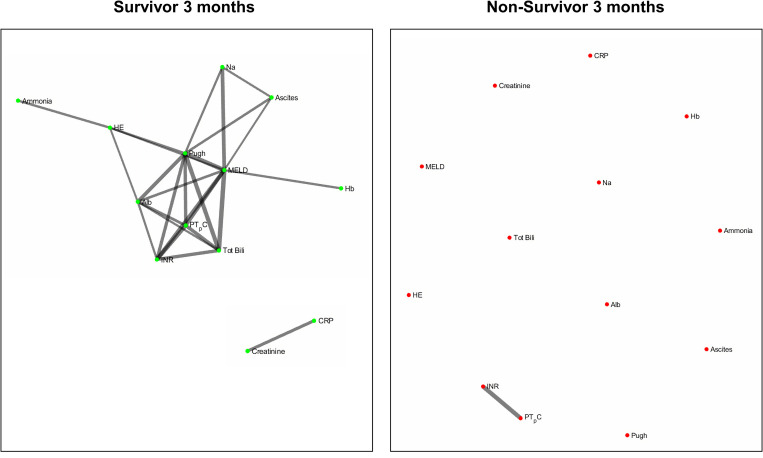
3-month Bonferroni corrected Pearson’s correlation analysis network map.

**FIGURE 3:**
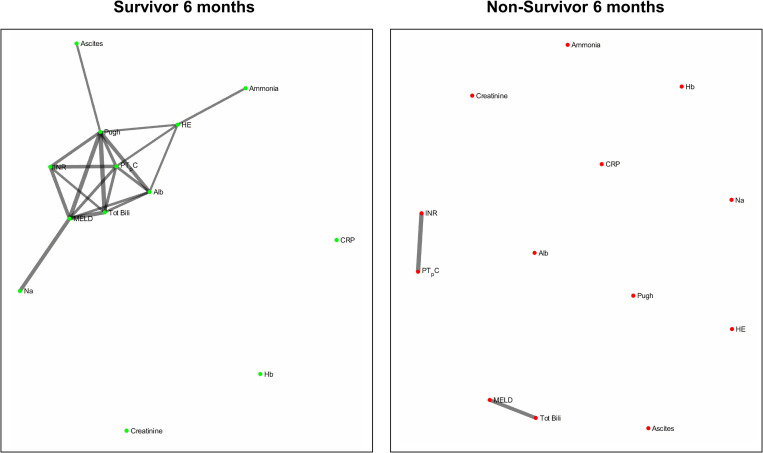
6-month Bonferroni corrected Pearson’s correlation analysis network map.

Figures 2 and 3 refers to the network structure of the 3- and 6-month Bonferroni corrected Pearson’s correlation analysis. There is a clear central cluster in survivors consistently throughout both months, with a lack of any substantial clusters in non-survivors.

**FIGURE 4:**
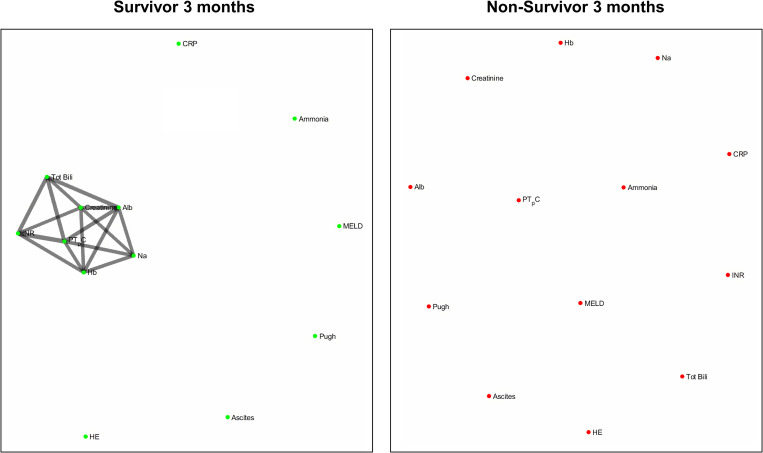
3-month mutual information analysis network map.

**FIGURE 5:**
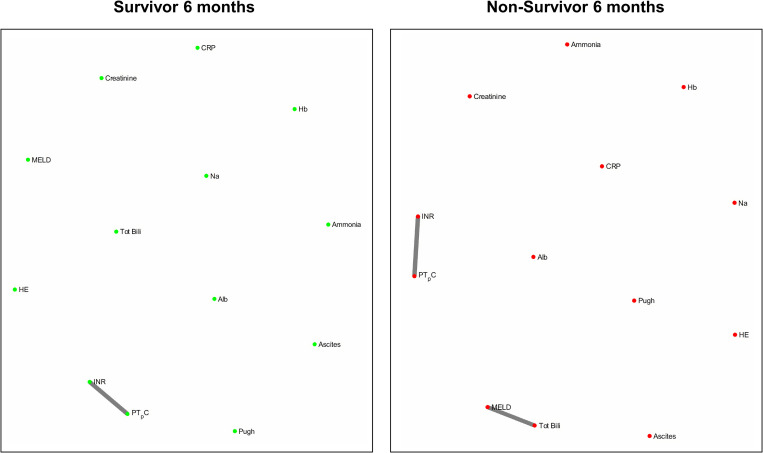
6-month mutual information analysis network map.

Figures 4 and 5 refers to the network structure of the 3- and 6-month mutual information analysis. In survivors, there is a central cluster of high interconnectivity in the 3 month network, but this cluster fails to materialize in the 6 month network. Conversely in non-survivors, there are no substantial clusters that are detected.

**FIGURE 6:**
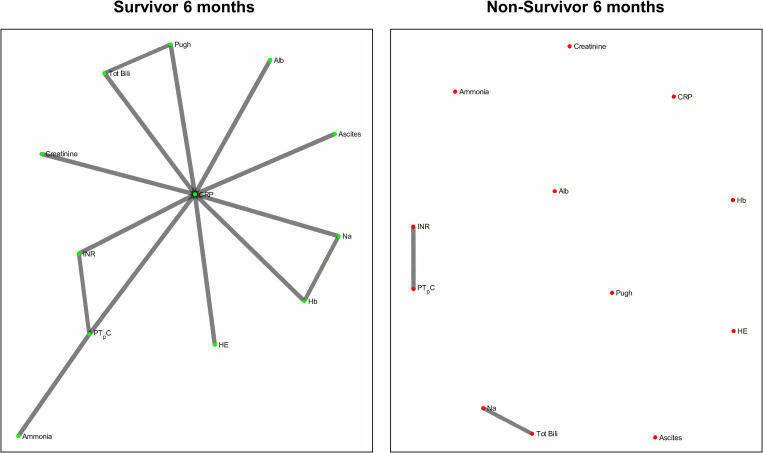
6-month pair-matched Bonferroni corrected Pearson’s correlation analysis network map.

Figure 6 refers to the network structure of the 6-month pair-matched Bonferroni corrected Pearson’s correlation analysis. In survivors, there is a substantial central cluster with edges branching out of the central node CRP. Conversely in non-survivors, there are no substantial clusters that are detected.

**FIGURE 7:**
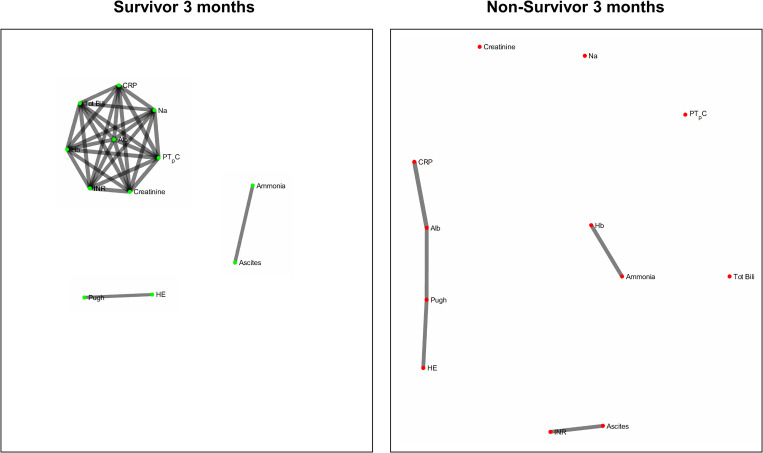
3-month pair-matched mutual information analysis network map.

Figure 7 refers to the network structure of the 3-month pair-matched mutual information analysis. There is a central cluster of high interconnectivity in survivors compared to a lack of network in non-survivors.

Overall, the network structure in survivor groups show consistent central clustering with high connectivity, in line with the overall higher number of edges compared to the non-survivor groups. Quantification of the networks are covered in the next section. The network structure for all follow-up times (3-, 6-, and 12-month) can be found in Supplementary Appendix B.

### Network Analysis Quantification

The network parameters measured for each network map in the general analysis is summarized in Table 3 for Bonferroni corrected Pearson’s correlation analysis, mutual information analysis, and the respective pair-matched analysis.

**TABLE 3:** Network parameters for **(A)** correlation analysis, **(B)** mutual information analysis, **(C)** pair-matched correlation analysis, and **(D)** pair-matched mutual information analysis.

		No. of edges	Average degree of connectivity	Average closeness	*t*-test *p*-value (degree)	*t*-test *p*-value (closeness)
**(A) Bonferroni corrected Pearson’s correlation analysis**						
3 months	Survivors	26	4.00	0.0383	1.10E-04	1.44E-06
	Non-survivors	1	0.15	0.0011		
6 months	Survivors	20	3.08	0.0293	1.28E-03	8.26E-05
	Non-survivors	2	0.31	0.0021		
12 months	Survivors	12	1.85	0.0193	3.26E-02	8.46E-03
	Non-survivors	5	0.77	0.0070		
**(B) Mutual Information analysis**						
3 months	Survivors	16	2.46	0.0182	1.53E-03	1.45E-03
	Non-survivors	0	0.00	0.0000		
6 months	Survivors	1	0.15	0.0011	1.86E-01	1.86E-01
	Non-survivors	2	0.31	0.0021		
12 months	Survivors	0	0.00	0.0000		
	Non-survivors	0	0.00	0.0000		
**(C) Pair-matched Bonferroni corrected Pearson’s correlation analysis**						
3 months	Survivors	0	0.00	0.0000		
	Non-survivors	0	0.00	0.0000		
6 months	Survivors	14	2.33	0.0494	9.38E-03	1.73E-09
	Non-survivors	2	0.33	0.0028		
12 months	Survivors	3	0.50	0.0041	0.5	0.5
	Non-survivors	3	0.50	0.0041		
**(D) Pair-matched mutual information analysis**						
3 months	Survivors	30	5.00	0.0413	2.22E-04	2.81E-04
	Non-survivors	5	0.83	0.0079		
6 months	Survivors	15	2.50	0.0321	3.39E-04	1.93E-06
	Non-survivors	6	1.00	0.0112		
12 months	Survivors	6	1.00	0.0106	1.55E-02	7.75E-03
	Non-survivors	2	0.33	0.0028		

## Discussion

The novel approach of organ system network analysis seeks to elucidate the complex mechanisms underlying life-threatening diseases of multisystem nature, in recognition that current linear clinical scores can be improved upon in assessing and reflecting (Bartsch et al., 2015; Holder and Clermont, 2015). By choosing clinical variables to represent organ systems, our study evaluated the differences in organ system connectivity between survivors and non-survivors of a group of 201 liver cirrhosis patients.

Our results demonstrate that organ system interaction was overall significantly higher in survivors compared to non-survivors, quantified by the total number of edges, average degree of connectivity and average closeness (Table 3). These findings support the hypothesis that decreased organ system interaction is associated with poor prognosis in chronic liver failure. Our findings also continue to support previous studies suggesting that systemic dysfunction in acute life-threatening pathophysiology with multisystem involvement is attributed to a loss of homeostatic interorgan connectivity (Buchman, 2002; Chovatiya and Medzhitov, 2014), most notably in the recent studies published by Asada et al. (2016, 2019). Although our study explores multisystem disease of a chronic nature, the characteristics associated with poor prognosis in non-survivors remain similar, namely the breakdown of organ system connectivity, loss of homeostatic stability and isolation of individual organ system clusters. The results of this study validate the use of a network approach in previously unexplored multisystem disease of a chronic nature and continues to highlight the importance of organ system network analysis in evaluating systemic stability of patients to improve prognostic outcome. However, it must be duly noted that this conclusion only applies to patients who have followed up and thus have a confirmed outcome at the end of the study. Patients with censored data (i.e., unknown outcome) may harbor bias within the network outcome by having a specific phenotype that results in their leaving of the study.

Current survival prediction and analysis scores such as the MELD-Na and Child-Pugh score for liver cirrhosis patients and their room for improvement in accountability of the complex mechanisms of multisystem disease encourages the consideration and investigation of alternate approaches such as the network approach. This is especially important as these scores form the basis of clinically important decisions such as the allocation of organ transplantation priority. Indeed, there has been a gradual shift toward a network approach where the original MELD score’s failure to account for specific pathophysiology and their effects on mortality risk prompted the transformation of the MELD score into the MELD-Na score (Xiol et al., 2009; Martin and O’Brien, 2015). Still, it is recognized that the extension of this clinical score to include renal biomarkers still leaves room to account for the systemic pathophysiology of liver cirrhosis. Some studies have suggested the addition of further predictive components such as in the proposal of the MELD-Plus score (Kartoun et al., 2017), while other studies have identified physiomarkers independent to the MELD score in mortality prediction. Montagnese et al. (2015) has shown that the addition of an EEG-based hepatic encephalopathy index to MELD improves prognostic accuracy, while Bhogal et al. (2019) has defined two heart rate variability indices that predict mortality in cirrhosis patients independently to MELD. The liver remains the primary site of initial pathophysiology development in cirrhosis, and though it is important to include specific liver biomarkers in survivor analysis, the progression of cirrhosis and its downstream effects means that inclusion of involved organ system biomarkers is paramount to reflecting the true complexity of liver cirrhosis.

The use of the Pearson’s correlation and mutual information analysis represent two approaches to defining the association strength between variables. Pearson’s correlation performs well under the assumption of linearity, but is widely used as the general correlation analysis index to elucidate preliminary information about a new dataset (Mukaka, 2012). In this study, such an approach is useful for achieving a general overview of the basic links within the network. Mutual information, as previously specified, accounts for the quantity of biological information transmitted independent of network complexity, and is sensitive to non-linear relationships (Steuer et al., 2002; Rhee et al., 2012). As biological networks operate in a dynamic and non-linear manner, the mutual information analysis presents a more robust measure in discerning and evaluating the true network structure of organ systems. In present study we observed that although both correlation and mutual information analyses exhibited higher network connectivity in survivors, some connections could only be detected by mutual information analysis. For example, hemoglobin level does not seem to be an important part of the correlation network in patents with cirrhosis while mutual information analysis indicates that hemoglobin is a hub in the survivor group and shares mutual dependence with other clinical variables such as serum albumin, creatinine, sodium and markers of blood coagulation. This finding may shed lights on possible adaptive mechanisms in cirrhosis which cannot be easily discovered using simple linear analyses such as Pearson’s correlation.

It is a possibility that the differences in networks between survivors or non-survivors could have been attributed to differences in the degree of liver cirrhosis severity across both groups, such that non-survivors might have an overall higher degree of disease severity. To correct for this, we carried out an analysis with pair-matching patients for MELD-Na scores. In the outcome of network graphs, there is a clear difference in the network structure between non-pair-matched and pair-matched analyses. Furthermore, the structural evolution of the networks over time also differs between non-pair-matched and pair-matched analyses. With regards to the Bonferroni corrected Pearson’s correlation analysis, the 6-month network structure of the pair-matched data (Figure 6) yields the most information as opposed to the 3 month network structure of the non-pair-matched data (Figure 3). From this, we can conclude that the degree of severity of disease is not the only driver of prognosis as the networks evolve.

We also sought to qualitatively analyse the clinical significance behind notable interactions seen on the network maps of patients. Such clinical significance can be expressed in the scope of validated literature of known pathophysiology. However, it is important to highlight that some of the interactions between clinical variables are obvious and expected, such as the [MELD-Na – total bilirubin] link and the [INR – prothrombin time] link, as these variables are closely related with a degree of dependency.

Of clinical significance are the difference in interactions of CRP in the network maps of survivors and non-survivors and the role of systemic inflammation. The survivor group maintains integration of CRP within the central cluster, whereas this interaction is lost or heavily isolated in the non-survivor group (Figures 6, 7). Conclusively, survivors maintain a link to the inflammatory and immune system, while non-survivors experience uncoupling of systemic inflammation from organ systems. CRP is an acute inflammatory protein synthesized primarily in the liver and is a key component in systemic inflammation (Sproston and Ashworth, 2018). Systemic inflammation is associated with disease progression in liver cirrhosis and patient mortality, accompanying the transition from compensated to decompensated cirrhosis, otherwise known as the systemic inflammatory response syndrome (SIRS) (Girón-González et al., 2004; Mahassadi et al., 2018). SIRS is triggered by systemic and life-threatening insults to the body (Balk, 2014).

Like many other life-threatening conditions such as sepsis, liver cirrhosis patients typically observe two phases associated with systemic inflammation. The first phase involves an increase in circulating cytokines, notably IL-6 and TNF-receptor expression (Tilg et al., 1992; Le Moine et al., 1994), which result in increased production of acute phase proteins such as CRP (Meliconi et al., 1988; Sheldon et al., 1993). This increase in circulating cytokines is largely attributed to hepatocyte death and bacterial translocation from increased intestinal permeability as a consequence of portal hypertension (Albillos et al., 2014; Arroyo et al., 2014; Bruns et al., 2014; Jalan et al., 2014). SIRS is hence characteristically observed in decompensated cirrhosis patients regardless of the presence of bacterial infection and is further associated with complications including hepatic encephalopathy and variceal bleeding (Shawcross et al., 2004; Thabut et al., 2007; Cazzaniga et al., 2009). SIRS acts in the capacity of a compensatory mechanism in maintaining systemic stability in the presence of other dysfunctional systems against life threatening illness (Gabay and Kushner, 1999; Asada et al., 2019).

The second phase occurs in the form of immune exhaustion, and in the context of liver cirrhosis is known as cirrhosis-associated immune dysfunction (CAID) syndrome (Albillos et al., 2014). CAID syndrome manifests through excessive activation of the inflammatory and immune system and loss of liver immune surveillance, resulting in non-responsiveness to immune challenges, endotoxin tolerance and immunodeficiency (Tiegs and Lohse, 2010; Giannelli et al., 2014; Sipeki et al., 2014). Given the homeostatic role of the liver in systemic immunity, progressive loss of liver function can consequently lead to failure in regulating the inflammatory response (Christou et al., 2007). Examples of such mechanism involve the breakdown of gp130-STAT3 signaling in hepatocytes and production failure of acute phase proteins. These components control the inflammatory response through mediation of autoregulatory myeloid-derived suppressor cells, an innate immune response in sepsis (Wong et al., 2005; Sander et al., 2010). The onset of a bacterial infection can be encouraged by the immunodeficient environment associated with CAID syndrome, or conversely it can push the onset of CAID syndrome. Regardless, the additional presence of bacterial infection increases the probability of mortality four-fold (Arvaniti et al., 2010) and likely represents the transition point of a patient into an uncontrolled septic state. This is significant as sepsis is responsible for 50% of deaths in cirrhosis patients (Wong et al., 2005). This transition point also likely reflects the loss of integration of the axis of inflammation with organ systems in non-survivors as a prognostic determinant, brought about by CAID syndrome in conjunction to bacterial infection. Consequently, we should expect to see the presence of peritonitis or infection in non-survivors.

The presence of highly interlinked central clusters observed in the network maps of survivors reflects successful homeostatic organ system connectivity, yet this is unlikely to be seen in normal, healthy people as increased organ system connectivity has been established as a compensatory mechanism to maintain homeostatic stability in preventing mortality during life-threatening illness (Gabay and Kushner, 1999; Asada et al., 2019). In fact, the network structures observed may indeed be a reflection of overcompensation, the same way the fight or flight reaction is all or nothing.

A correlation analysis is appropriate for generating the network map of a group of patients, given that multiple samples are available for each pair of variables being analyzed. However, this approach does not work with only a single patient, a shortcoming of such network analysis for feasible application in medicine. To make the network approach clinically viable, there must be a method to analyze the network of a single patient. This is made possible with a recently introduced network type, termed a parenclitic network, which makes it possible to represent a collection of isolated and heterogenous scalar values as a network (Zanin et al., 2014). The basis upon which this works is by calculating the deviation between the data of a single patient with the pre-constructed reference model to weight the link between corresponding nodes. This mathematical framework involving high level analysis is suitable regardless of relationship type or dataset, as long as features are numerical. With this approach, the network map of a single patient can be generated and analyzed mathematically, and with expert domain knowledge input, presents a viable method in evaluating multisystem disease.

Further models using a network approach should include more physiological data, such as heart rate variability and temperature variability indices. Reduced heart rate variability and body temperature variability are known to be inversely associated with increased mortality in cirrhosis patients, where their prognostic value is independent of MELD or Pugh scores (Mani et al., 2009; Bhogal et al., 2019; Bottaro et al., 2020). In a subset of patients investigated, the uncoupling of the autonomic nervous system and cardiovascular system was found to be associated with mortality in liver cirrhosis patients. Clinically, excessive inflammation during cirrhosis progression is known to facilitate cytokine-induced autonomic neuropathy and the uncoupling of the cardiac pacemaker regulation (Hajiasgharzadeh et al., 2011). While such a physiomarker adds immense clinical value to the model, further exploration is required to determine the optimal subset and number of clinical variables for organ system representation and network analysis. A wide range of feature selection methods exist that may be helpful in arriving at the optimal subset of clinical variables (Maniruzzaman et al., 2018) and should be further investigated. Future studies will benefit from including larger number of patients from different hospitals. The low number of patients used to construct the non-survivor networks presents as a limitation in our study. Our data cannot be generalized to all cirrhotic patients as our cohort of patient data comes from a single hospital and our conclusions can only apply to patients who have followed up throughout the length of the study.

## Conclusion

This is the first study to apply an organ system network analysis approach to a multisystem disease of chronic nature, providing quantitative and qualitative evidence of the role of systemic stability in patient outcome. Such an evaluation method of organ system interactions has high potential in clinical application for improving patient outcomes with proper implementation, given the room to improve within our current clinical score methods for decision making in the accountability of the complex pathophysiology of multisystem disease.

## Data Availability Statement

The raw data supporting the conclusions of this article will be made available by the authors, without undue reservation, to any qualified researcher. Requests to access these datasets should be directed to AM, a.r.mani@ucl.ac.uk.

## Ethics Statement

The studies involving human participants were reviewed and approved by University Hospital of Padova Ethics Committee. The patients/participants provided their written informed consent to participate in this study.

## Author Contributions

YT, SM, and AM contributed to the conceptualization. SM and YT contributed to the data curation. YT contributed to the formal analysis, software, and the writing of the original draft. SM and AM contributed to the supervision. YT, SM, and AM contributed to the writing – review and editing. All authors contributed to the article and approved the submitted version.

## Conflict of Interest

The authors declare that the research was conducted in the absence of any commercial or financial relationships that could be construed as a potential conflict of interest.
