# Supplementary material for: Organ System Network Disruption Is Associated With Poor Prognosis in Patients With Chronic Liver Failure
Source: Front Physiol. 2020 Aug 5;11:983. doi: 10.3389/fphys.2020.00983 (PMC7422730; doi:10.3389/fphys.2020.00983)
Supplement: Supplementary file 1 [file Table_1.DOCX]

# Appendix A

Pair-matching algorithm

1. Assign a column *X* as the pair-match criteria variable for samples.
2. Calculate the sample size for survivor and non-survivor datasets and assign the smallest dataset as the primary dataset to be pair-matched against. Let us denote this smallest dataset as *A* and the dataset to be matched against as *B*.
3. For *i*th sample in *A*, where *i* represents every sample iteration in the dataset *A* and *B_match_* represents a match to the sample *A_i_*, if there is:
   1. 1 exact match, assign *A_i_* to output matrix instance *C_i_* and assign *B_match_* to output matrix instance *D_i_*, and remove *A_i_* and *B_match_* from *A* and *B*.
   2. More than 1 exact match, assign *A_i_* to output matrix instance *C_i_* and randomly select 1 of the possible *B_match_* choices and assign *B_match_* to output matrix instance *D_i_*, and remove *A_i_* and *B_match_* from *A* and *B*.
   3. No exact match, expand the pair-match criteria range by ±0.5 or an otherwise specified value. For *i*th sample in *A*, if there is:
      1. 1 exact match within the specified range, assign *A_i_* to output matrix instance *C_i_* and assign *B_match_* to output matrix instance *D_i_*, and remove *A_i_* and *B_match_* from *A* and *B*.
      2. More than 1 exact match within the specified range, assign *A_i_* to output matrix instance *C_i_* and randomly select 1 of the possible *B_match_* choices and assign *B_match_* to output matrix instance *D_i_*, and remove *A_i_* and *B_match_* from *A* and *B*.
      3. No exact match, assign a blank value to output matrix instance *C_i_* and output matrix instance *D_i_*, and remove *A_i_* from *A*.
4. Remove all instances of empty values from *C* and column *X*, designating *C* and *D* as pair-matched datasets.
