# Supplementary material for: Organ System Network Disruption Is Associated With Poor Prognosis in Patients With Chronic Liver Failure
Source: Front Physiol. 2020 Aug 5;11:983. doi: 10.3389/fphys.2020.00983 (PMC7422730; doi:10.3389/fphys.2020.00983)

## SEPARATE DOCUMENT

## Bonferroni Corrected Pearson’s Correlation network analysis


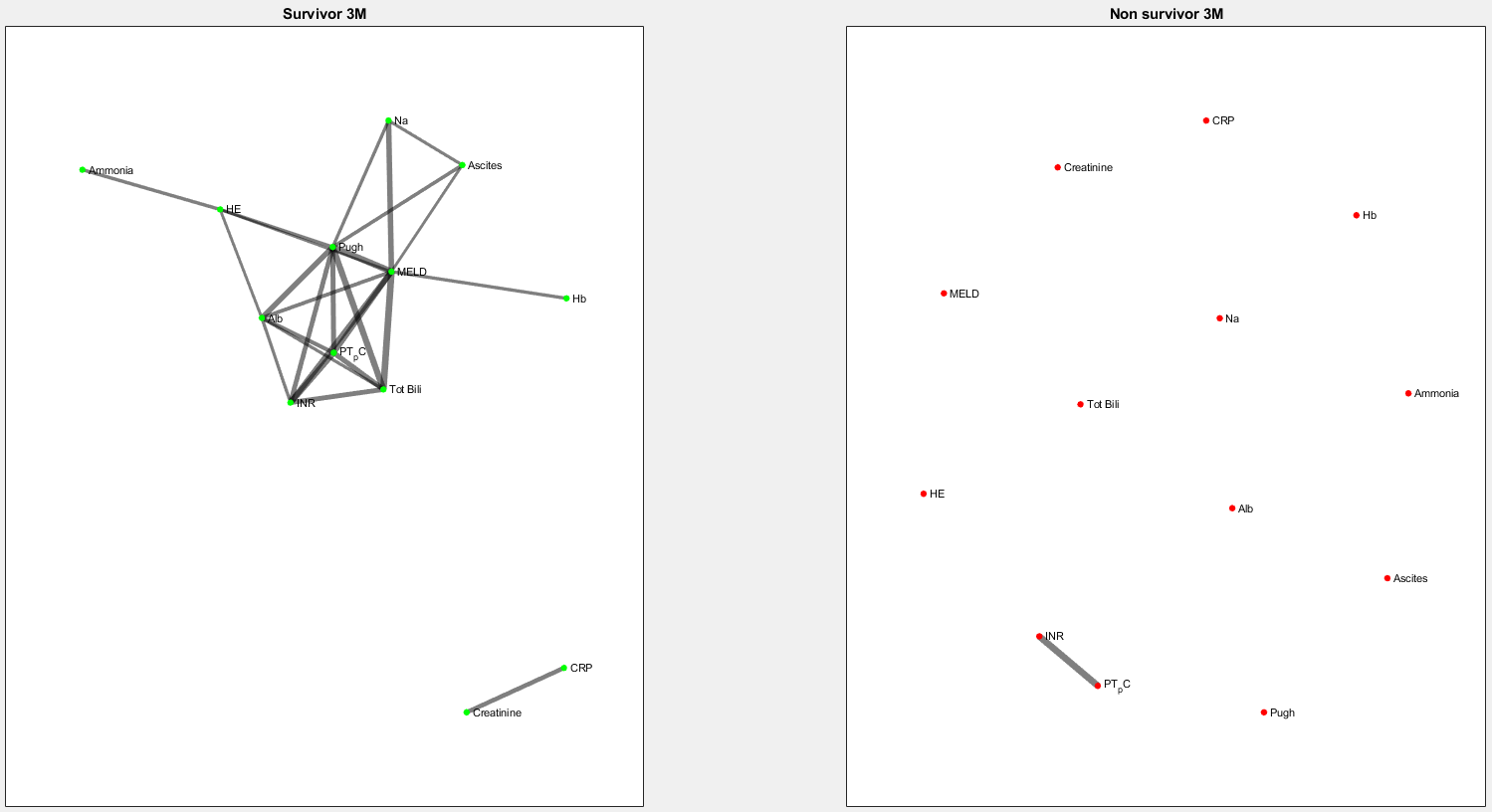


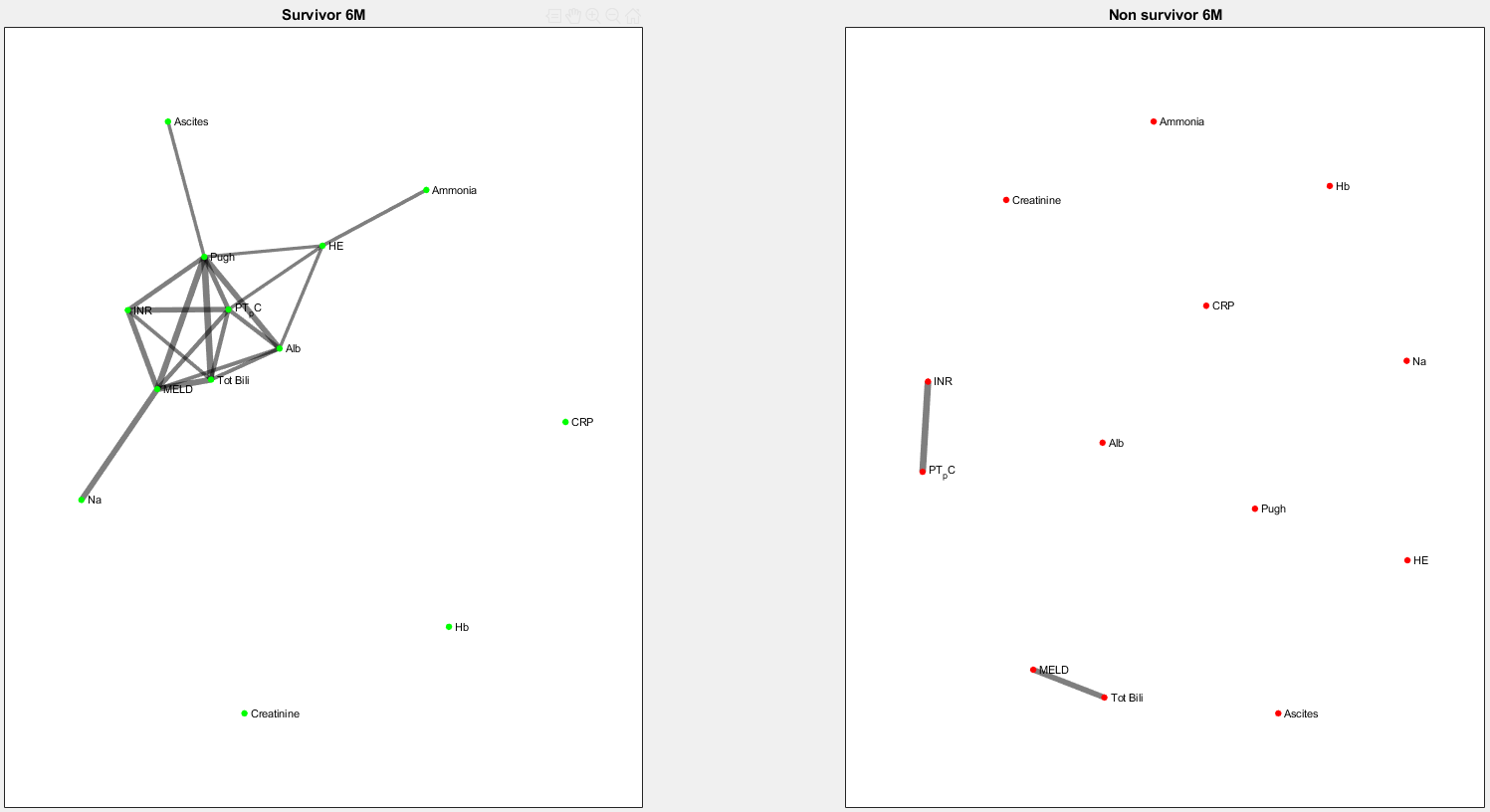


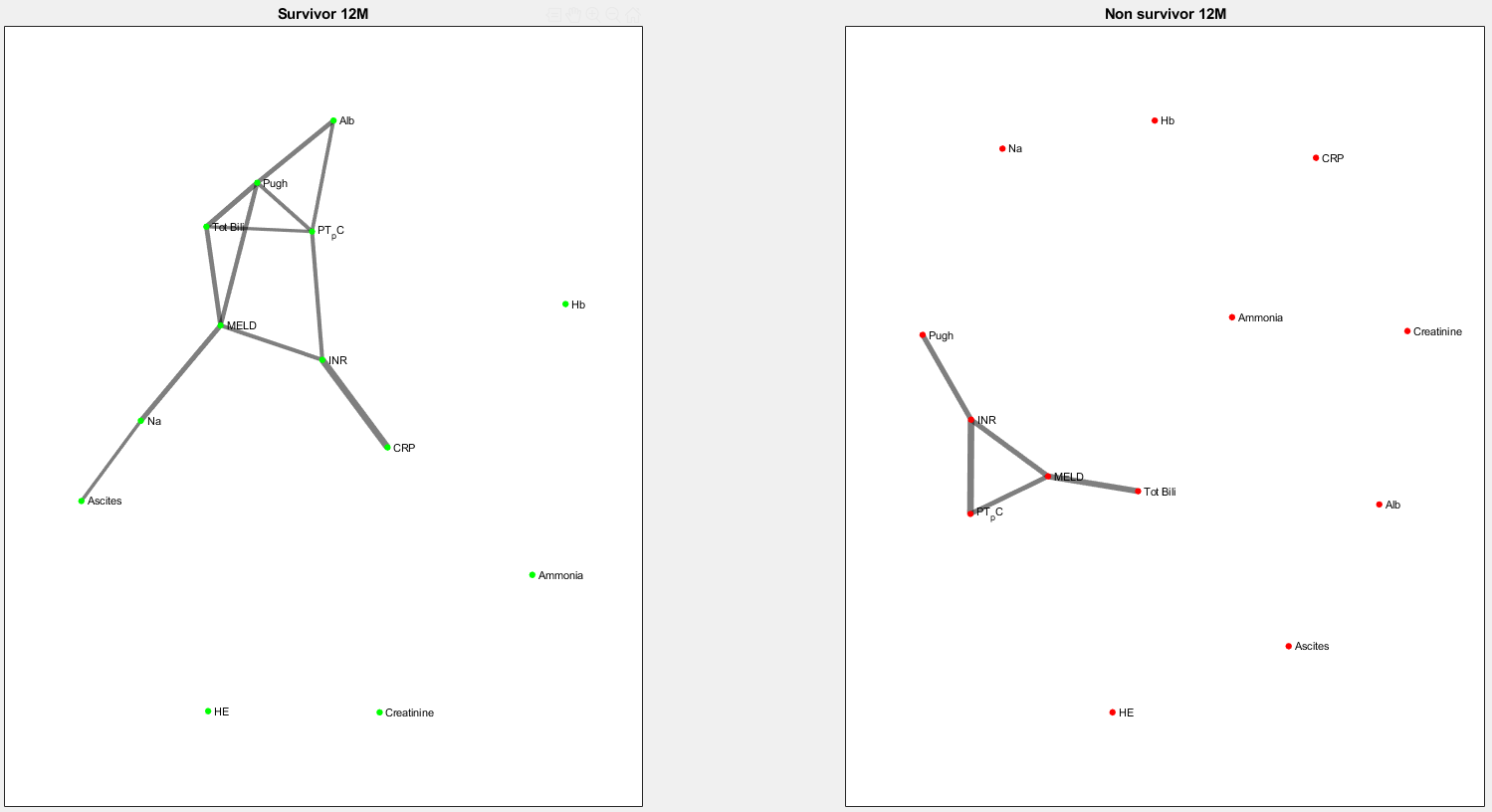


## Mutual Information network analysis


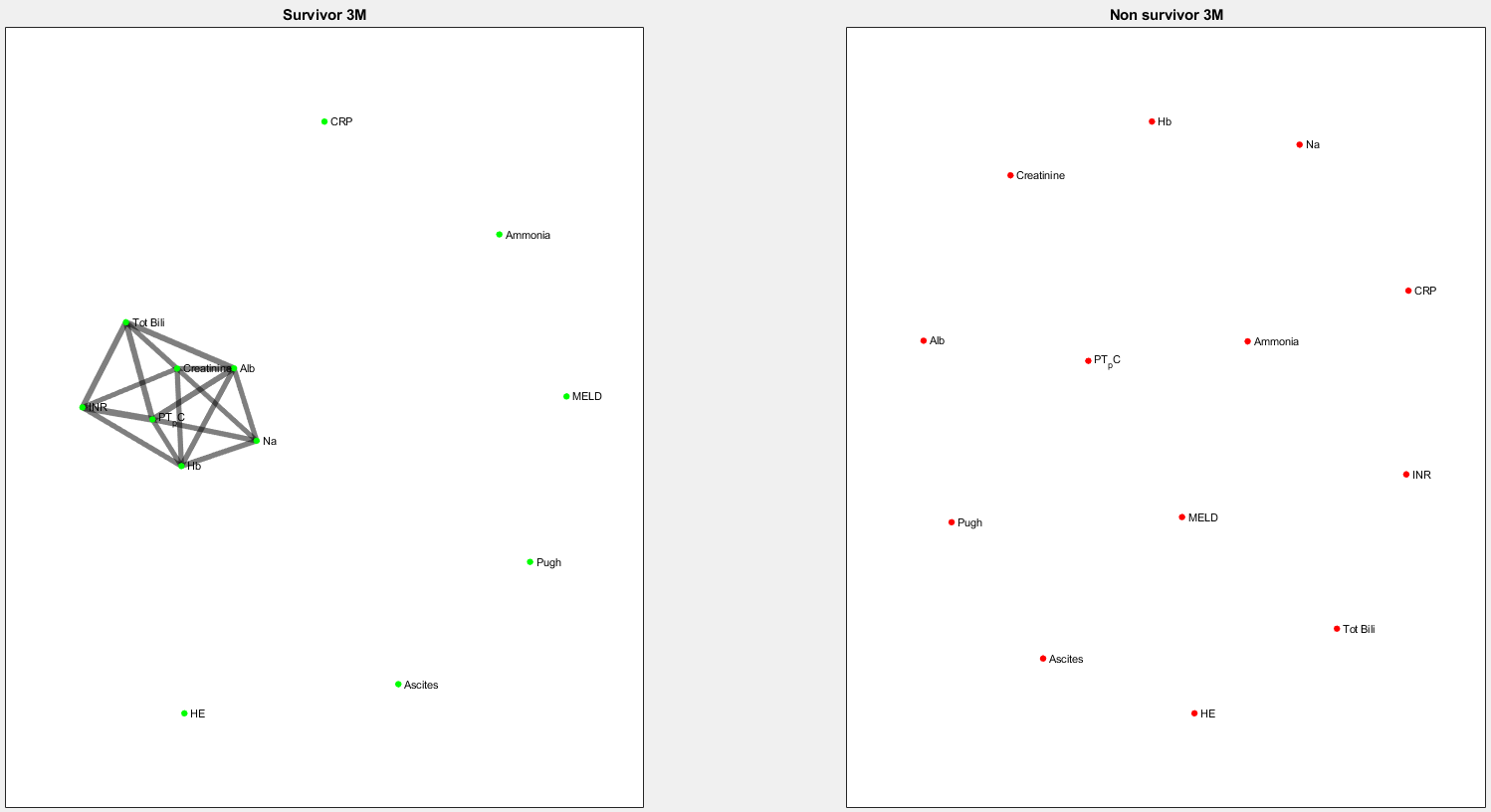


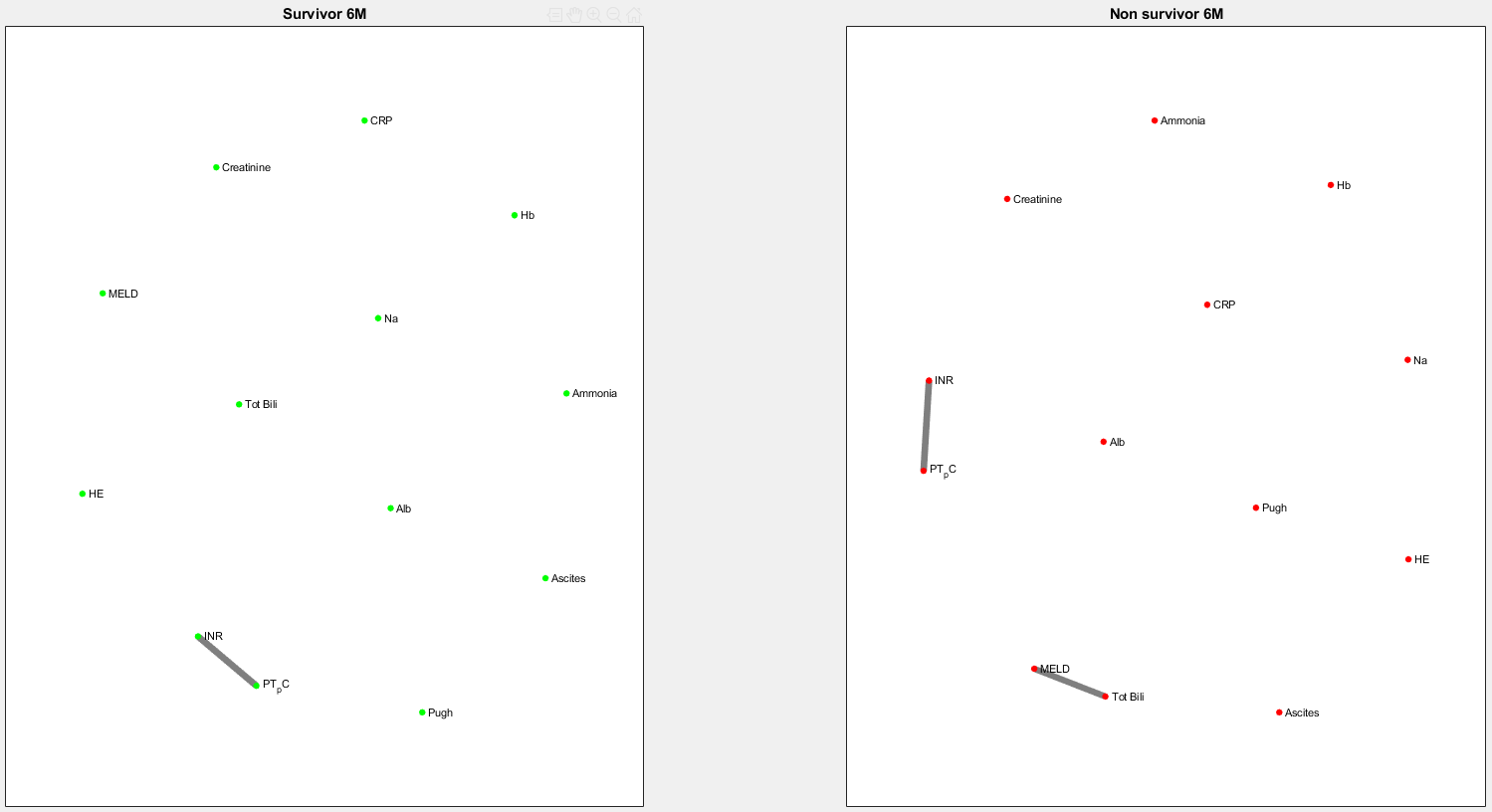


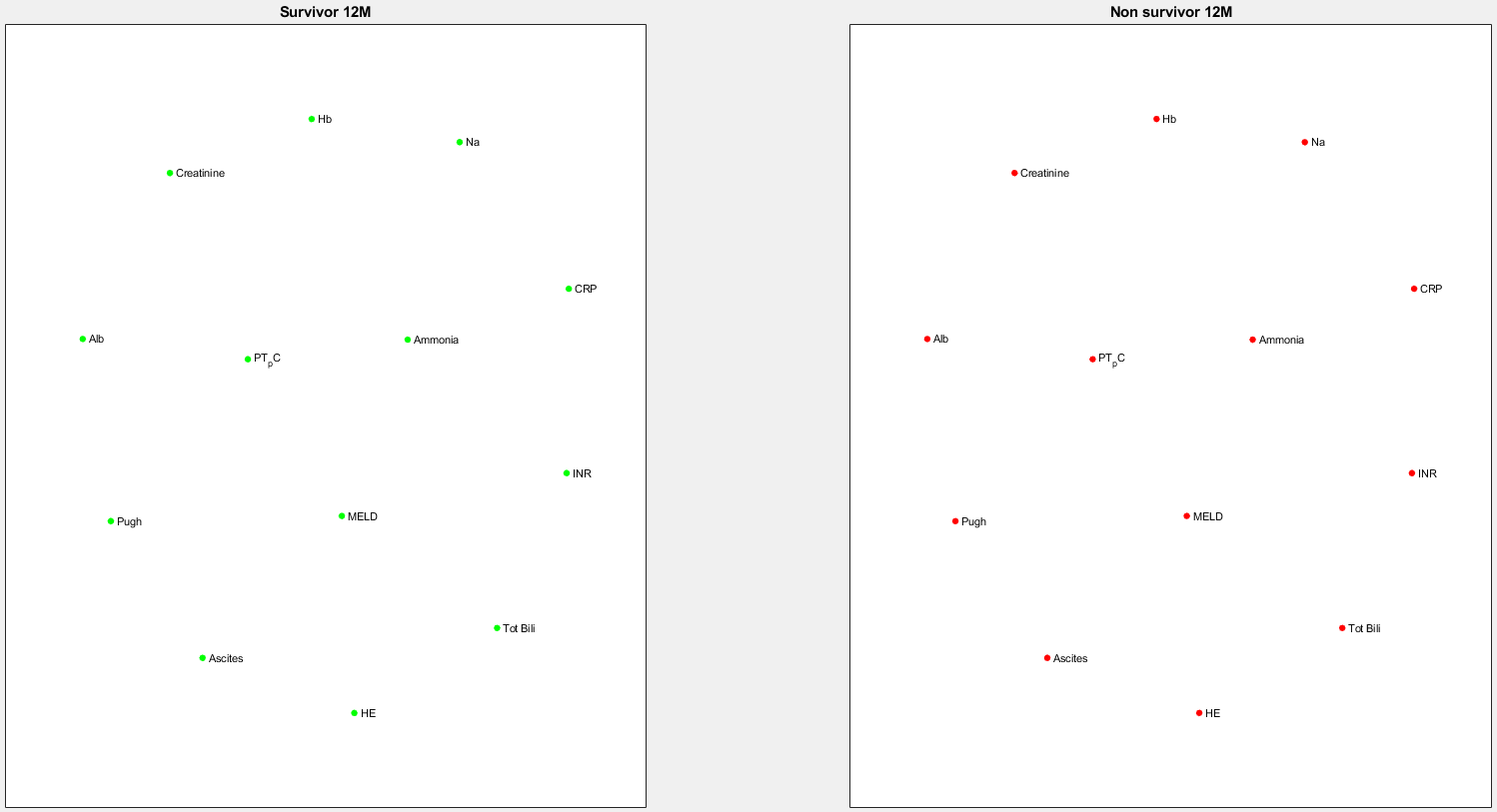


## Pair-matched Bonferroni Corrected Pearson’s Correlation network analysis


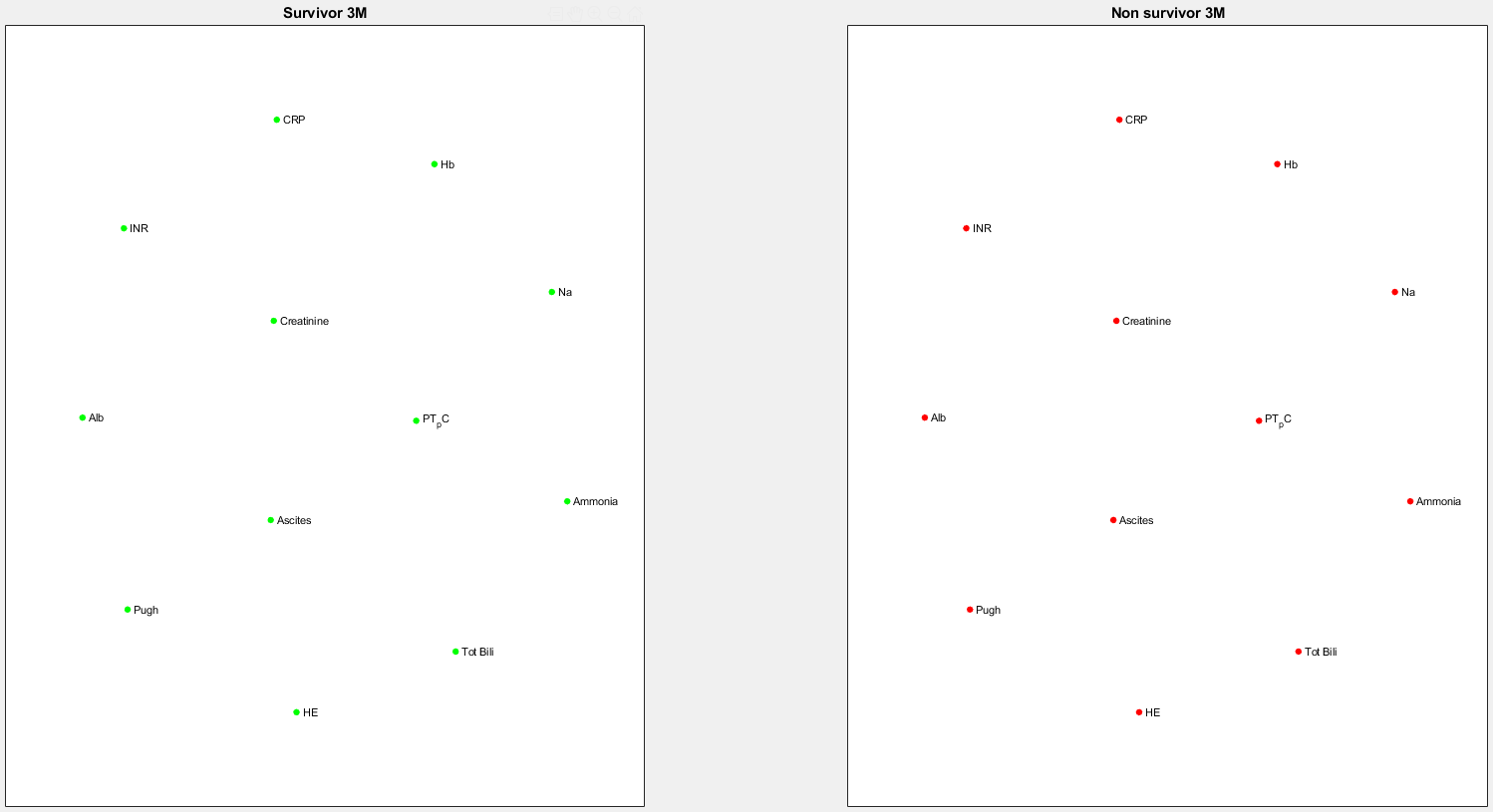


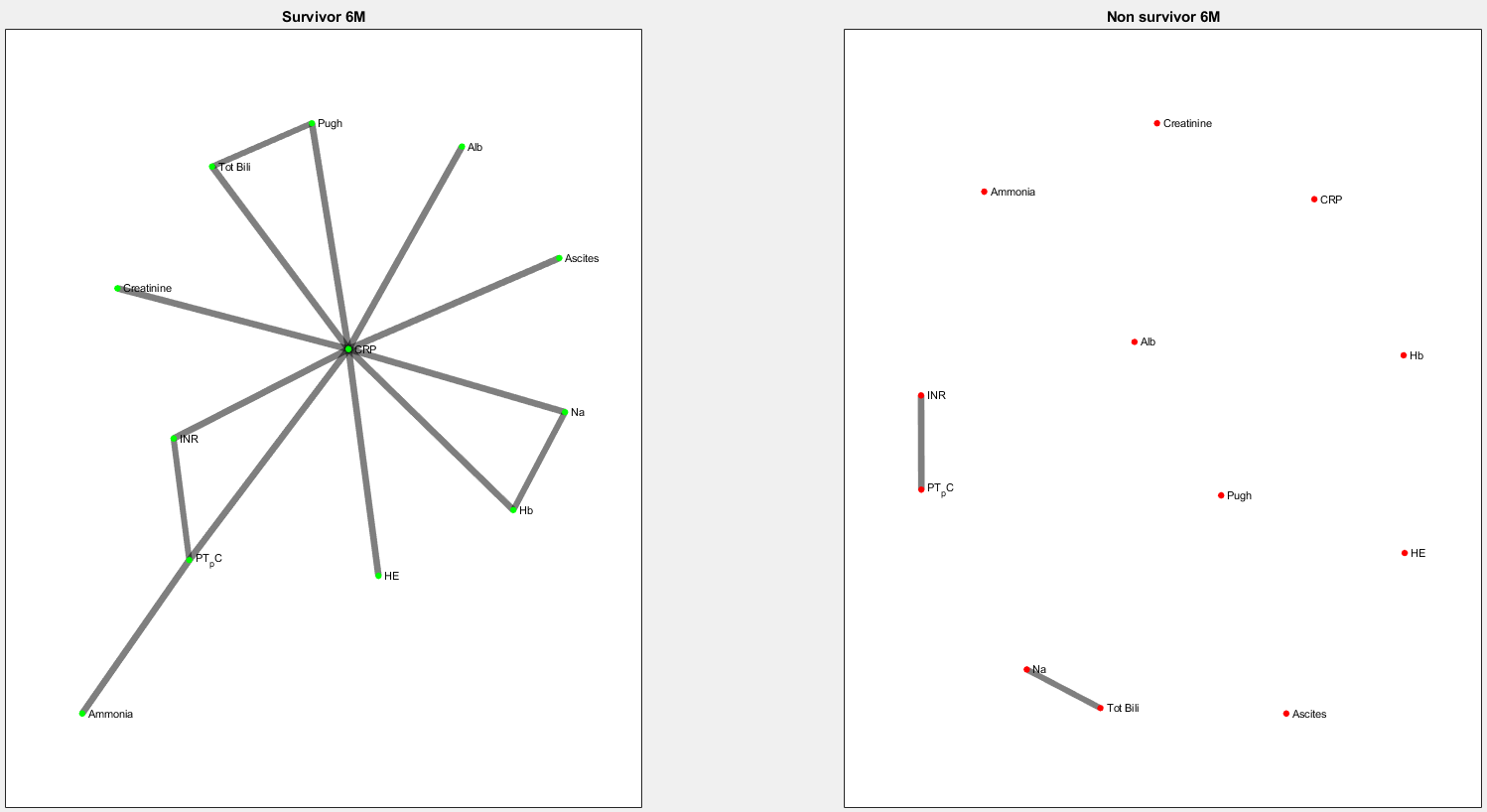


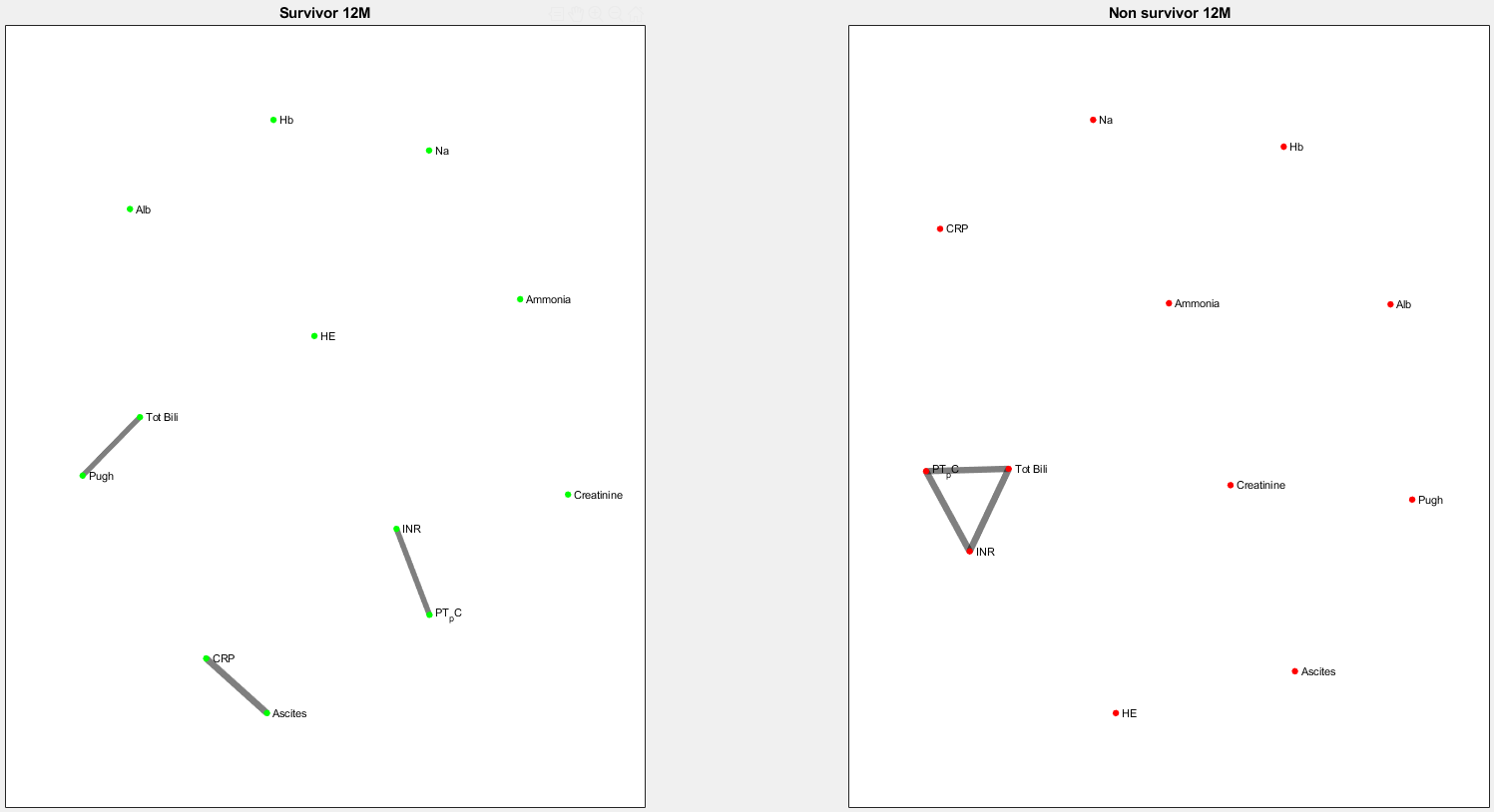


## Pair-matched Mutual Information network analysis


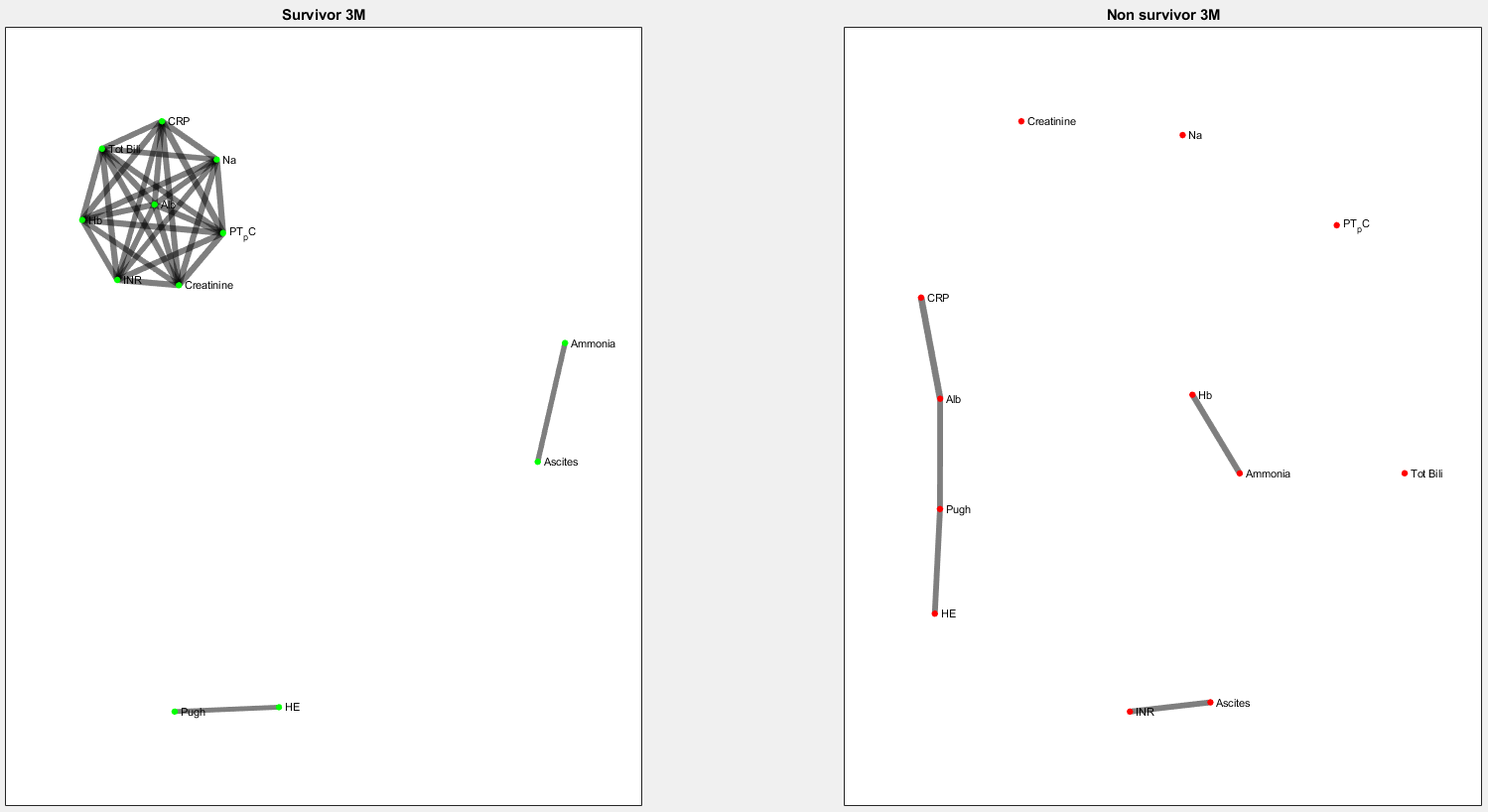


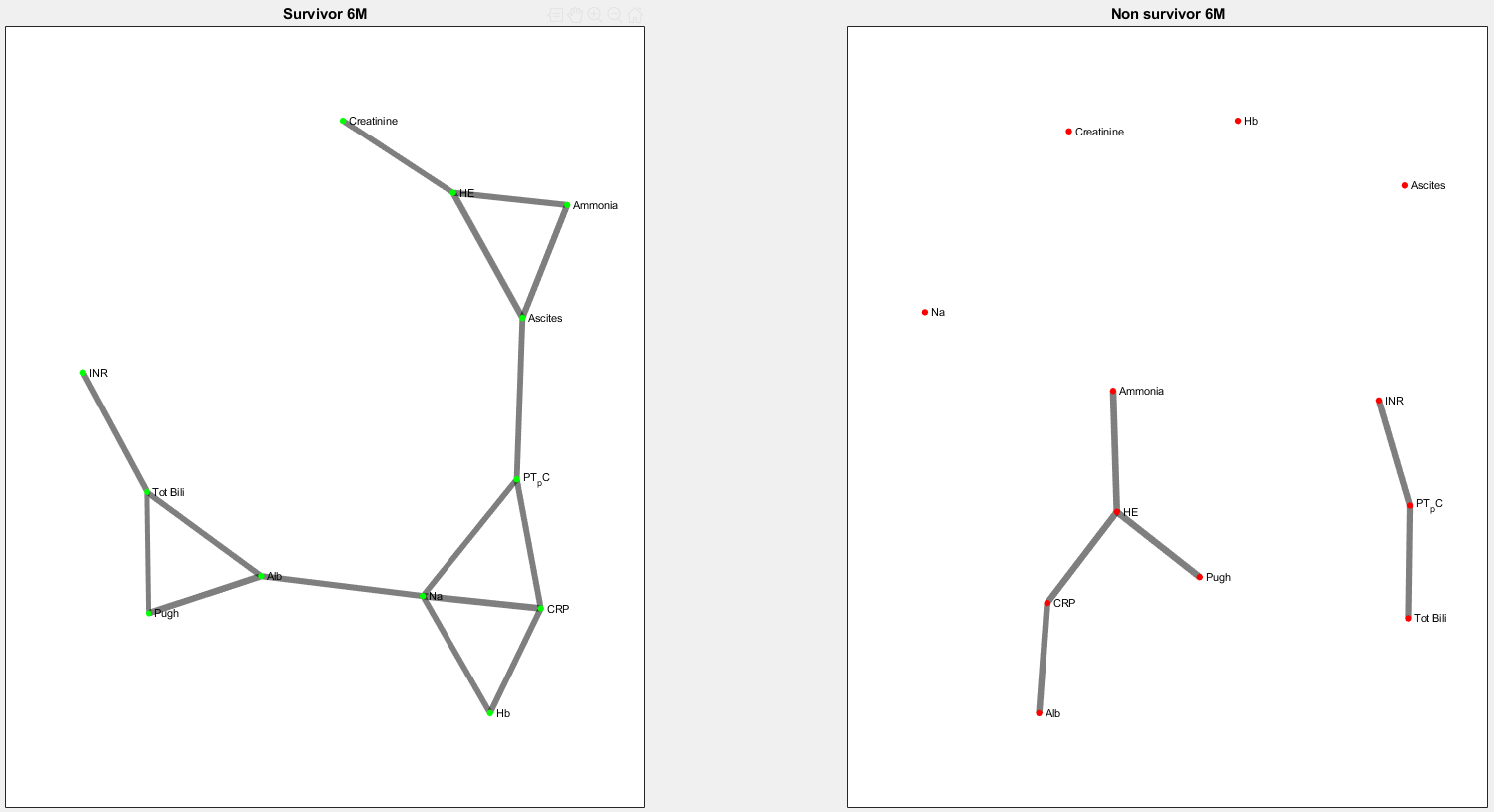


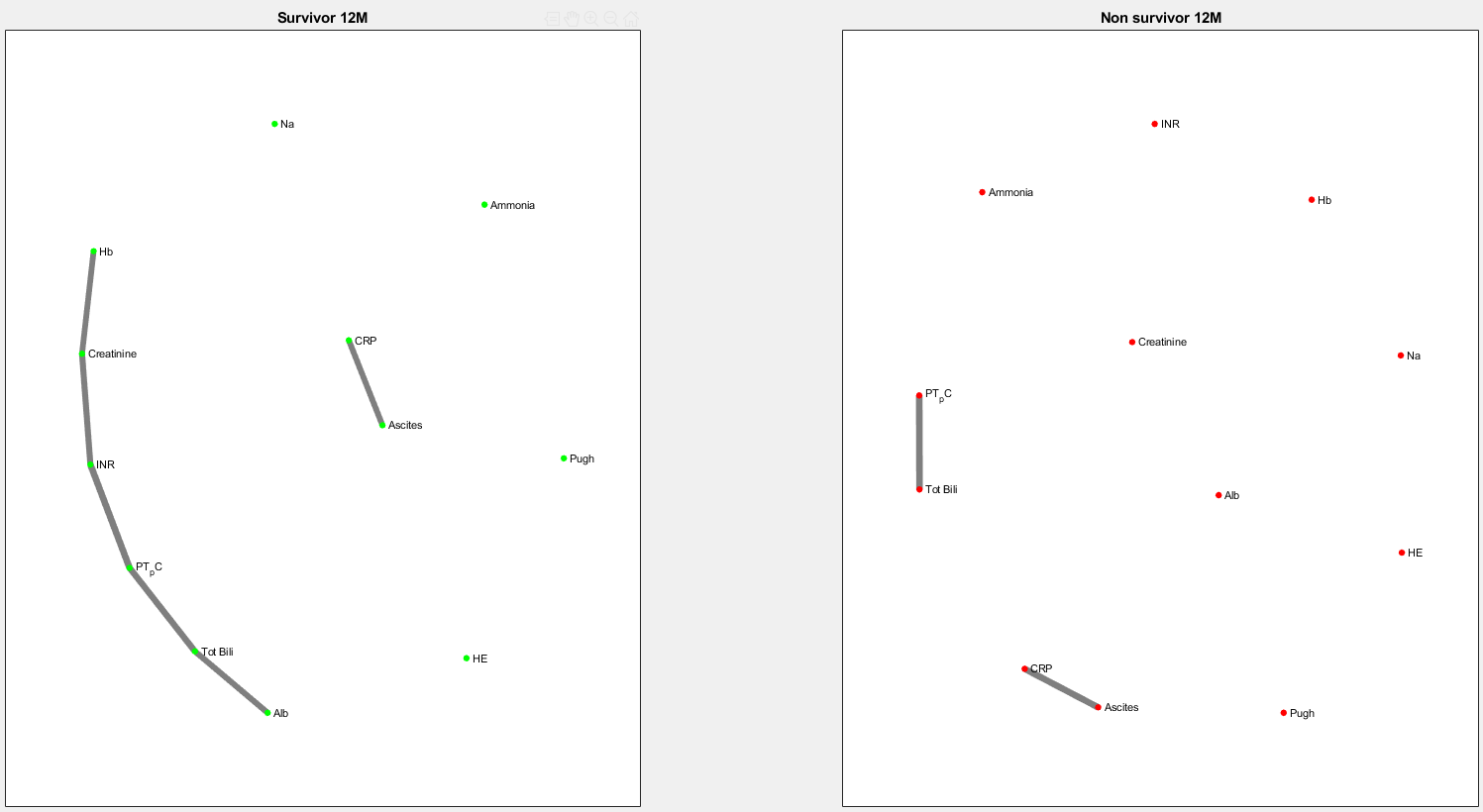

Supplement: Supplementary file 2 [file Table_2.DOCX]
